# The involvement of perivascular spaces or tissues in the facial intradermal brain-targeted delivery

**DOI:** 10.1080/10717544.2019.1587044

**Published:** 2019-03-31

**Authors:** Wei Yang, Bing-Hui Jin, Ya-Jing Chen, Chang Cao, Jia-Zhen Zhu, Ying-Zheng Zhao, Xi-Chong Yu, Fan-Zhu Li

**Affiliations:** a College of Pharmaceutical Science, Zhejiang Chinese Medical University, Hangzhou, China;; b School of Pharmaceutical Sciences, Wenzhou Medical University, Wenzhou, Zhejiang Province, China

**Keywords:** Perivascular spaces or tissues, trigeminal nerve, brain, lymphatic network, facial intradermal brain-targeted delivery, mystacial pad, blood-brain barrier

## Abstract

Our previous work indicates the lymphatic network and perivascular spaces or tissues might be involved in the facial intradermal brain-targeted delivery of Evans blue (EB). In this article, we presented the detailed involvement of both, and the linkage between lymphatic network and perivascular spaces or tissues. The *in-vivo* imaging, the trigeminal transection and immunohistochemistry were used. *In-vivo* imaging indicated intradermal injection in the mystacial pad (i.d.) delivered EB into the brain at 2-, 6- and 24 h, while intranasal injection (i.n.) delivered EB into the rostral head and intravenous injection (i.v.) diffused EB weakly into the brain. Trigeminal perineurial and epineurial EB occurred along the perivascular spaces or tissues and along brain vessels. EB diffused into the lymphatic vessels and submandibular lymph nodes. Moreover, perineurial and epineurial EB co-located or overlaid with Lyve1 immuno-reactivity and VEGF antibody, and lymphatic network connected with perivascular spaces or tissues, suggesting lymphatic system-perivascular spaces might involve in the EB delivery with i.d. The trigeminal transection reduced the trigeminal epineurial and perineurial EB and brain EB along vessels. EB diffused in the fasciculus and the perineurium, blood and lymphatic vessels in the mystacial pad, mystacial EB overlaid VEGF or Lyve1 antibody. In summary, the dermal-trigeminal-brain perivascular spaces or tissues and the linkage to the lymphatic network mediated the intradermal brain-targeted delivery.

## Introduction

The facial brain-targeted delivery might be an alternative for bypassing the BBB. Although brain-targeted delivery systems facilitated the drug delivery to the brain (Miyake and Bleier, 2015; Zeiadeh et al., 2018; Wong et al., 2019), the blood-brain barrier (BBB) remains handicap to the systemic drug delivery to the brain with insufficient improvement of CNS diseases (Pathan et al., 2009; Miyake and Bleier, 2015). The intracerebral and intrathecal injection overcome the BBB with brain injury (Griffin, 2008; Shi et al., 2011; Paul et al., 2015), while intranasal brain-targeted delivery benefits CNS diseases with lower delivery efficiency and without injury. We previously reported that facial intradermal brain-targeted delivery, an alternative strategy for bypassing the BBB via several trigeminal sub-structures with unclear mechanisms, is more efficient than the intranasal brain-targeted delivery with minimally invasive injury of facial skin (Yu et al., 2017), indicating the promising strategy for bypassing the BBB.

The perivascular spaces or tissues might be pathways for facial intradermal brain-targeted delivery. Lacking lymphatic networks, brain parenchymal perivascular spaces and tissues functioned as a glymphatic system or pathways for CSF drainage and substances diffusion, such as the Aβ protein and blood components (Rennels et al., 1985; He et al., 2012; Yin et al., 2013; Gallina et al., 2015; Morris et al., 2016; Plog and Nedergaard, 2018). On the other hand, perivascular spaces or tissues served as the pathways for penetrations of melatonin and the fluorescence tracers into the brain (Yang et al., 2013; Reiter et al., 2014), indicating the possible roles of perivascular spaces or tissues in brain-targeted delivery. Indeed, the nasal perivascular spaces functioned as a rapid pathway for intranasal delivery to brain (Dhuria et al., 2010; Lochhead et al., 2015). With facial intradermal brain-targeted delivery, Evans blue (EB) occurred along the outmost layer of trigeminal vasculatures and perivascular tissues in the perineurium, epineurium and dura, and nasal-brain pathways involved in the delivery (Yu et al., 2017), implying trigeminal, brain and nasal perivascular spaces or tissues were involved in facial intradermal brain-targeted delivery.

The dermal-trigeminal-CNS lymphatic systems might connect to the perivascular spaces or tissues. In the CNS, dural lymphatic vessels, perivascular spaces, and parenchymal paravascular microcirculation function as exchange channels, and the dural lymphatic vessels connected the perivascular spaces and the extra-cranial lymphatic systems (Durant and Yaksh, 1986; Iliff and Nedergaard, 2013; Rangroo Thrane et al., 2013; Aspelund et al., 2015; Maloveska et al., 2018). The trigeminal perineurium and the epineurium elongate from pia and dura mater, respectively. Trigeminal perineurium has microcirculatory beds and lymphatic vessels (Smoliar et al., 1998; Furukawa et al., 2008). Trigeminal perivascular spaces and lymphatic network involved in the facial intradermal brain-targeted delivery. Moreover, dermal lymphatic system mediated the dermal drug absorption, including the facial intradermal brain-targeted delivery (Jain et al., 2008; Harvey et al., 2011; Yu et al., 2017). It suggested the dermal-trigeminal-CNS lymphatic network connected to perivascular spaces or tissues in facial intradermal brain-targeted delivery. In the present research, we evaluated the role of perivascular spaces or tissues with linkage to dermal-trigeminal-CNS lymphatic network in the facial intradermal brain-targeted delivery.

## Methods and materials

### Materials

Evans blue (EB) was purchased from Sigma Aldrich and dissolved in double-distilled water (0.71% for rats and 0.5% for mice). VEGF monoclonal antibody and Lyve1 polyclonal antibody were purchased from Abcam, USA and Santa Cruz Biotechnology, USA, respectively.

### Animals

Male ICR mice, 20–22 g, and male Sprague–Dawley Rats, 200–240 g, were provided by the Animal Center of Wenzhou Medical University. Animals were housed a specific-pathogen-free (SPF) breeding facility with 12:12 light-dark cycle at 26° C and 50 ± 5 percentage humidity. Animals were free to access the food and water. Rats or mice were fasted without water deprivation for 12 h before the test. All experimental protocols and procedures were approved by the Animal Care and Use Committee of Wenzhou Medical University (Ethical No. wydw2014-0089).

### Intranasal delivery and intradermal injection in the mystacial pad

The intradermal, intravenous or intranasal injection was described previously (Yu et al., 2017). For intranasal injection (i.n.), briefly, being anesthetized with isoflurane (2.5% isoflurane, with 80% oxygen and 20% room air), the mouse lay on upright 45 degrees-supine with tracheal cannula. Evans blue(0.5%) at 10 μL/10 g(body weight) was injected via PE10 tube at 1 μL/min. The same dose of EB intradermal injection in the left mystacial pad (i.d.) and intravenous injection (i.v.) via the tail vein were performed. Also 0.71% 10 μL/100 g (body weight) was injected intradermally in rat left mystacial pad. After i.d. and i.v., rat and mouse kept the same position as i.n. for 20 min.

### *In-vivo* animal imaging

In the pre-experimental, we did not get clear images of rat head with or without EB due to the thick skull of rat blocked the red fluorescence of EB, therefore we used the mouse for in-vivo animal imaging. After anesthesia with 300 mg/kg chloride hydrate, the mouse hair was removed with depilatory paste (human, made in Japanese) and cleaned carefully with 50% alcohol. The mouse was housed alone for recovery from skin stimulation for 2 days. EB i.d., i.n. or i.v. as described above. At 2h-, 6h- and 24h-post injection, the mouse was transferred into the image system (Maestro^TM^ with 2.10 Software, USA) and put in the prone, ventral or lateral position. Its head was exposed to the optic camera directly. The excitation and emission filter were set to get the fluorescence of EB (Excitation wavelength at 620 nm and emission wavelength at 680 nm). The images were acquired and calculated by the metro software. At 6 h-post injection, mice those received i.d., i.n. and i.v. were sacrificed, brains, and trigeminal nerves were dissected for *in-vivo* imaging after cardio-perfusion with 50 mL saline.

### Trigeminal transection

The rat was placed in the lateral position after the anesthesia with 2.5% isoflurane. The maxillary nerve, a main trigeminal branch innervated the mystacial pad, was exposed with blunt dissection. The rostral maxillary nerve near the mystacial pad was ligated with two sutures and then the maxillary nerve between two sutures was cut off. The wound was sutured and EB i.d. was performed. Six hours post EB i.d., the rat was sacrificed and the caudal maxillary nerve was prepared for fluorescence observation.

### Immuno-histochemistry and –fluorescence

At 6 h post-i.d. injection, the rat brains and trigeminal nerves were isolated after cardio-perfusion with 4% paraformaldehyde (dissolved in 0.1 M PB). The trigeminal nerve or brain blocks were dehydrated by 30% sucrose (dissolved in 0.1 M PB) until the sinking. The brain blocks and trigeminal nerves were buried in O.C.T and 5 μm-frozen sections were prepared. The section was incubated with 3% H_2_O_2_ (contains 80% methanol) to inactivate the endogenous peroxidase for 10 min. After being rinsed with 0.01 M PBS 5 min × 3, the section was blocked by 5% goat serum for 60 min. The primary antibody, Lyve1(1:100) antibody or VEGF(1:400) was added to the section and incubated at 37° C for 2 h. HRP-conjugated secondary goat anti-rabbit IgG covered the section for 1 h at 37° C after 0.01 M PBS rinse. The section was stained by DAB and hematoxylin for immuno-histochemistry, or 90% glycerol covered the sections for fluorescence observation. For EB fluorescence observation, the section was rinsed with 0.01 M PBS for 10 min, and covered by 90% glycerol.

## Results

### Intradermal injection enhanced EB diffusion into the mouse brain

**Figure 1.**
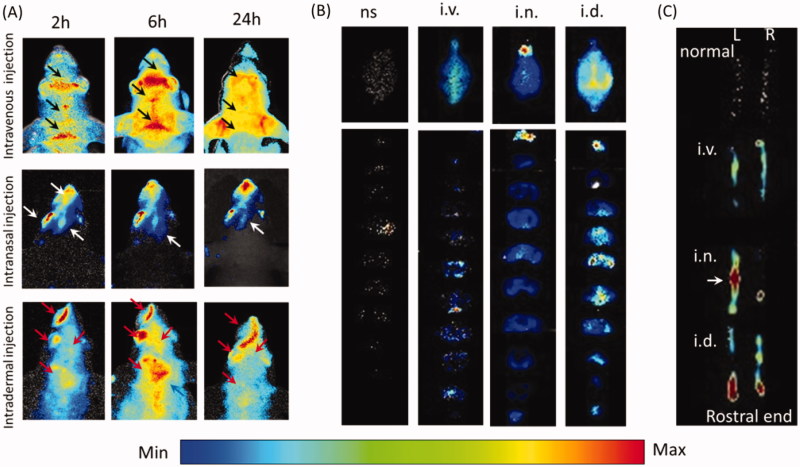
Intradermal injection enhanced Evans blue diffusion into the brain. (A) In-vivo images of the mouse head after intranasal, intravenous and intradermal injection of Evans blue. (B) Images of the whole brains and the corresponding slices from intravenous, intranasal and intradermal injection of Evans blue. (C) Images of right and left trigeminal nerves with i.d., i.n. and i.v. of Evans blue injection. ns: normal; i.v.: intravenous injection via tail vein; i.n.: intranasal injection; i.d.: intradermal injection in the mystacial pad.

In-vivo imaging indicated EB distribution in the mouse head varied from different administration routes. Two hours and 6 h-post i.v., EB shaped like the venous sinus in the head and EB evenly diffused into head at 24 h (Figure 1(A), black arrows). After i.n., EB occurred in the rostral head, including the nose, ipsilateral eye and the rostral brain at 2, 6 and 24 h, and EB faded away from 6 h (Figure 1(A), white arrows). With i.d., head EB accumulated and peaked at 6 h and then decreased at 24 h. EB concentrated in the nose, caudal head and neck instead of the whole head (Figure 1(A)), red arrows). Head EB with i.d. was more intense than with i.n or i.v., and shaped differently, implying EB might penetrate into the brain after i.d. or i.n via different pathways. However, dermal EB might confuse with brain EB in *in-vivo* animal imaging.

Indeed, EB occurrence in the brain was evidenced by brain dissection. i.v. increased olfactory EB and along the middle line of the brain, EB mainly occurred in the ventral side of in middle brain slices (Figure 1(B)). i.n. enhanced the olfactory EB mainly (Figure 1(B)). With i.d., intensive EB occurred in the whole brain, especially along the middle line. In addition, i.d. showed higher EB in the caudal brain than other areas and those from i.n. or i.v. EB distributed in the caudal brain in the brain slices, particularly the brain stem (Figure 1(B)), indicating the i.d. delivered EB to the brain via olfactory bulb and brain stem. With i.d., left trigeminal nerve showed obvious dye in the rostral end, while i.n. enhanced middle trigeminal nerve and i.v. or non-injection increased slightly (Figure 1(C)). In consideration of the trigeminal innervation of the mystacial pad and the EB distribution in the ipsilateral caudal brain, EB in brain stem might come from the trigeminal nerve after i.d.

### EB with i.d. distributed into rat trigeminal vasculatures in the epineurium and perineurium

**Figure 2.**
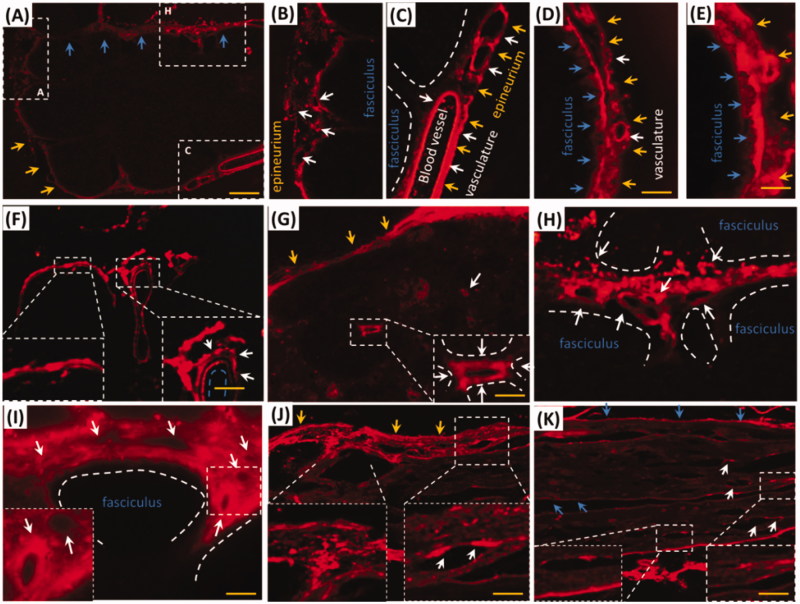
EB diffused into vasculatures and perivascular spaces or tissues in trigeminal epineurium and perineurium. (A) and (B) EB diffused in epineurium and perineurium; (C), (D) and (E) EB distributed in vasculatures of epineurium; (F) EB occurred around vessels and showed sandwich structure. (G)-(I) EB in trigeminal perineurium showed porous structures. (J) and (K) EB diffused along the perineurium and epineurium. Yellow arrows: epineurium; blue arrows perineurium; white arrows: vasculatures; the bar is 50 μm, (A) and (F) 10×; D), (E) and (G)-(K) 20×.

i.d. might deliver EB via trigeminal nerve, whether did EB with i.d. distribute into the trigeminal tissues of rat? As expected, EB distributed into trigeminal sub-structures, such as vasculature, fasciculus, epineurium and perineurium after i.d. EB diffused in trigeminal epineurium (Figure 2(A–E,I), yellow arrows) and the embedding vasculatures (Figure 2(A,C,D), white arrows). Besides the large vessels in the epineurium, EB diffused into the little vasculatures in rostral and caudal trigeminal epinurium (Figure 2(D,E), white arrows) and showed porous structures (Figure 2(B) white arrows), implying lymphatic or blood capillary and the surrounding tissues might be pathways for EB diffusion. Vascular EB shaped like sandwich structure, EB stained the endothelial cells and the outmost layer of vessels but not the middle layer of vessel (Figure 2(F), white arrows). EB also diffused into the trigeminal perineurium with gradient descent, and displayed porous as well as in the epineurium (Figure 2(A,G–I), white arrows). EB distributed along the trigeminal perineurium and epineurium in sagittal sections (Figure 2(J,K), blue arrows) and the EB-stained vasculatures scattered between fasciculus. It indicated trigeminal vasculatures and perivascular tissues were involved delivery from the mystacial pad.

### Trigeminal perivascular spaces and lymphatic network were involved in the delivery of EB with i.d

**Figure 3.**
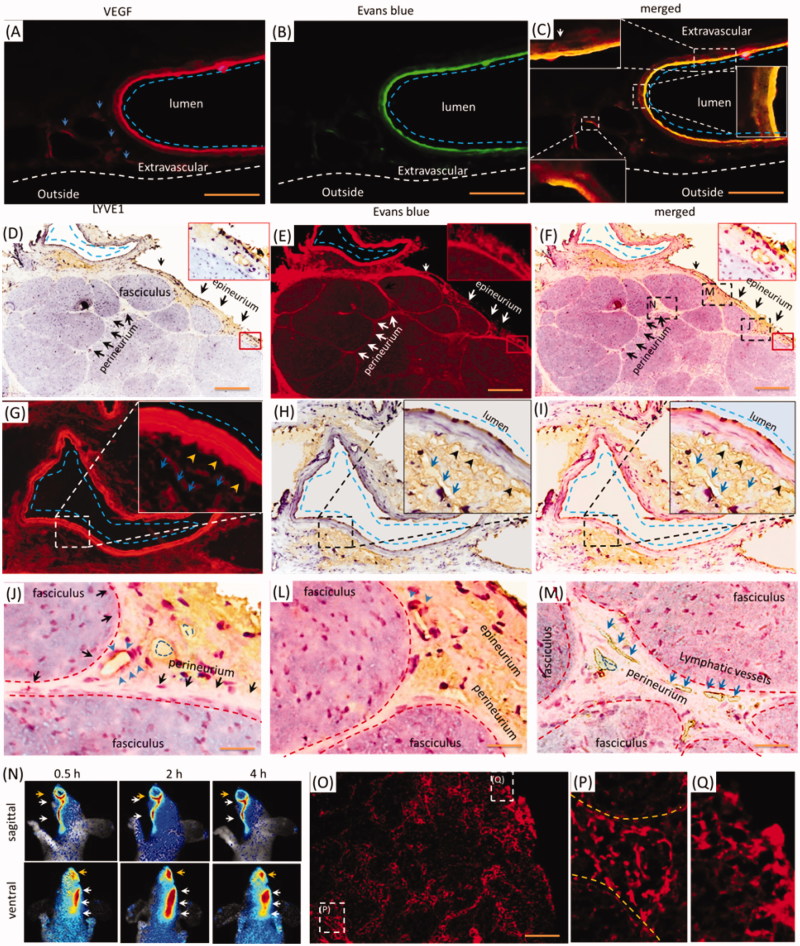
Trigeminal vasculatures and perivascular spaces were involved in the delivery of EB with i.d. (A–C) EB and Lyve1 distributed into trigeminal substructures; (D) Lyve1 expressed in the epineurium and perivascular tissues or tissues. (E) EB distributed along blood vessel and its surrounding tissues. (F) Trigeminal EB overlaid Lyve1 partly. (G) Lyve1 immuno-reactivity located near the blood vessel and connected to perivascular spaces, Lyve1 overlaid EB partly around the vessel. (J)-(M) Lyve1 overlaid epineurial and perineurial EB partly. (N) In-vivo living image of EB diffusion to lymphatic vessels and node. (O–Q) EB diffused into submandibular lymph nodes and lymphatic vessels. White or black arrows: perineurium, epineurium or lymphatic vessel; blue arrows or dash circles: lymphatic vessels; yellow arrows: perivascular spaces; blue arrows and dash line: blood vessel. The bar is 50 μm, (A–C) 10×; (D–F) and (N) 10×; (A–C), (G-I) and (O–Q) 20×.

As indicated in Figure 2, the porous structures in the perineurium and epineurium indicated blood vessels or lymphatic network might mediate the EB delivery with i.d. Vascular EB in endothelium was higher than in the outmost layer, and the perivascular also stained by EB weakly (Figure 3(A), blue dash line). EB overlaid the VEGF immune-activity in the epineurium (Figure 3(A–C), blue dash line), demonstrating the vasculatures and perivascular tissues were involved in the delivery of EB. Because VEGF-antibody also detected the lymphatic vessels, whether were lymphatic vessels or network involved in the i.d.? The Lyve1 immuno-reactivity located beside the epineurial blood vessel, but Lyve1 immuno-reactivity overlaid EB partly (Figure 3(D–I), blue dash line and arrows). Additionally, Lyve1 staining indicated the surrounding tissues of blood vessel structured porously (Figure 3(G–I)). It showed vessel-like structures and located beside the outmost layer of the vessel (Figure 3(G–I), blue arrows and black arrowheads), indicating the lymphatic network might link to perivascular spaces. Given the EB impermeability of the endothelium, trigeminal perivascular spaces or tissues connected lymphatic system and were involved in the delivery EB after i.d. In the trigeminal epineurium and perineurium, EB surrounded or located beside the Lyve1 immuno-reactivity and EB overlaid the interstitial tissue (Figure 3(D–F,J,L), blue arrows or blue dash line). Lymphatic vessels also occurred in the deep trigeminal perineurium but did not overlay EB (Figure 3(M)). Moreover, EB with i.d. occurred in the lymphatic vessels and submandibular lymph node (Figure 3(N–P)). Thus, the lymphatic network-perivascular spaces or tissues mediated the EB diffusion after i.d., and lymphatic network linked to perivascular spaces and functioned as pathways for EB diffusion.

### EB with i.d. occurred in brain perivascular tissue or spaces

**Figure 4.**
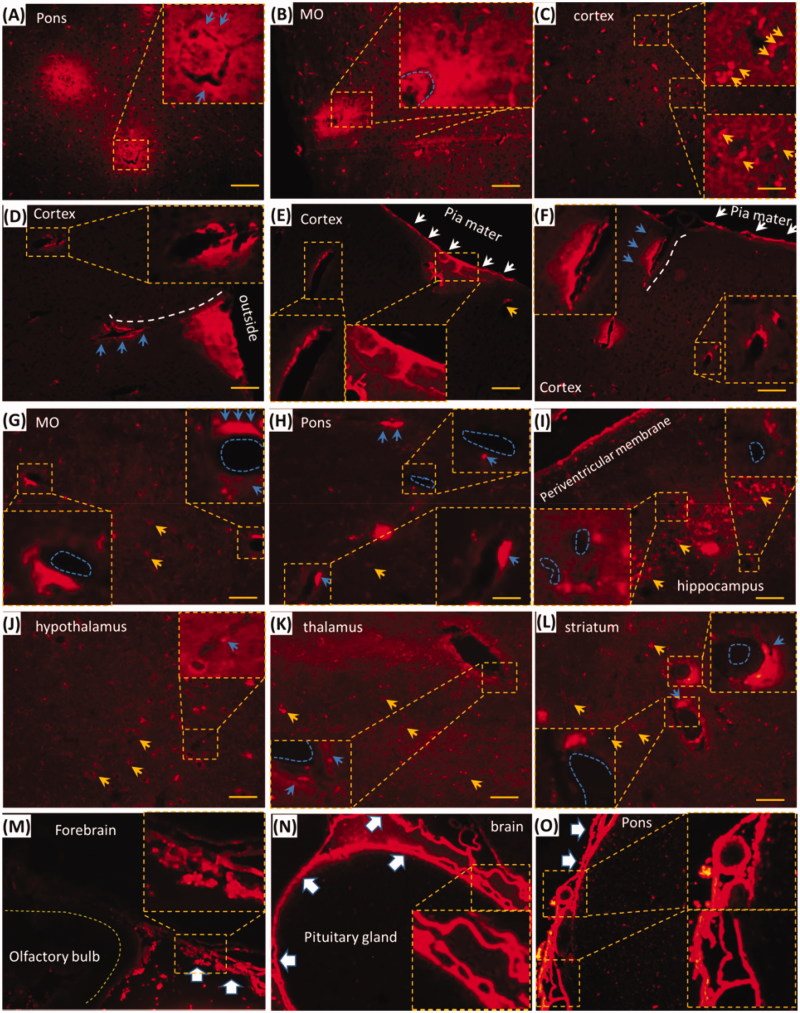
EB with i.d. diffused into brain perivascular tissues or spaces and the effects of PG on the brain EB. (A–C) EB diffused into the brain parenchyma without perfusion. (D–F) EB distributed around cortical blood vessel; (G–L) EB located near blood vessels in Pons, medulla oblongata, thalamus and hypothalamus, striatum and hippocampal CA2. (M–O) EB diffused into the dura and pia mater. Blue dash circle: blood vessels; Blue arrows: EB in the perivascular tissues or spaces; Yellow arrows: neurons; White arrows and dash line: EB in pia mater and along vessels. Big white arrows: dura and pia mater. Pons: pons varolii; MO: medulla oblongata. The bar is 50 μm, 20×.

Trigeminal lymphatic network-perivascular spaces or tissues might be pathways for EB delivery of i.d. (Figure 3) and EB diffused into the brain (Figure 1). Whether could EB distribute into the brain perivascular spaces or tissues? In the brain without perfusion, EB diffused around blood vessels and decreased gradually from the blood vessel (Figure 4(A,B), blue arrows or dash circle), while EB beside or along vessels was stronger than non-vascular tissues or cells, such as neurons (Figure 4, yellow arrows). In the brain with perfusion, EB occurred along or around vessels in the brain parenchyma (Figure 4(D–I)). Cortical EB located along vessels from the pia mater to the brain parenchyma (Figure 4(D–F)), white dash circles or arrow). Hippocampal, striatal, medullary, pontile, thalamic and hypothalamic EB occurred around or beside the vessels (Figure 4(H–L), blue dash circles and arrows). It indicated that EB with i.d. could be delivered to the brain via the vasculature, perivascular spaces along the vessels. Dural and leptomeningeal EB shaped porous structures, including vasculatures, and it was intense than the brain parenchymal EB (Figure 4(M–O), big white arrows), suggesting the involvement of dural and leptomeningeal vasculature in i.d. delivery. Given the linkage between the lymphatic network and perivascular spaces and the involvement of perivascular spaces in intranasal delivery to the brain, the lymphatic network mediated EB delivery after i.d. to the brain via perivascular spaces of tissues.

### Trigeminal transection reduced trigeminal and brain EB along perivascular spaces or tissues

**Figure 5.**
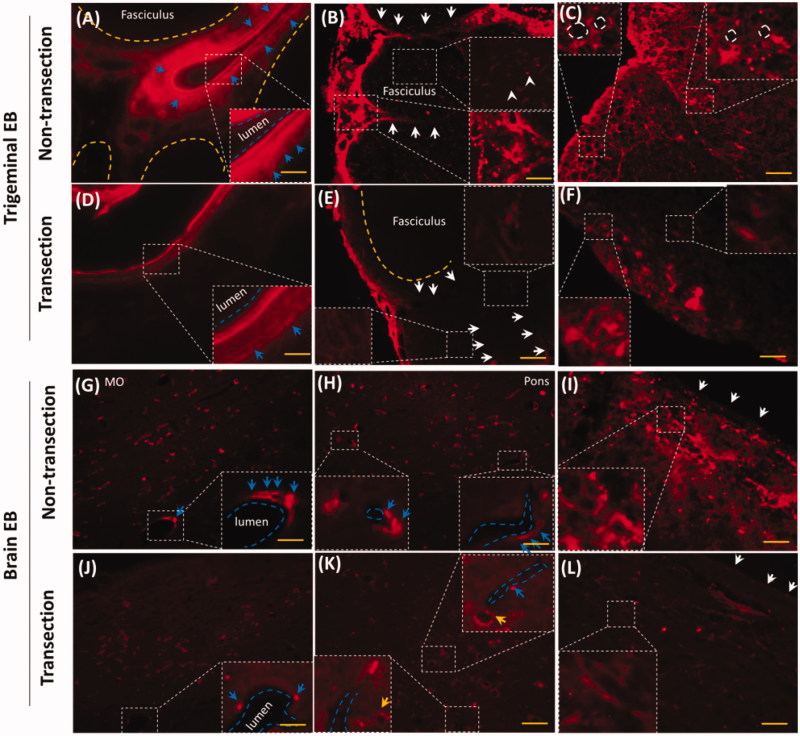
Trigeminal transection reduced brain and trigeminal perivascular EB. (A–C) EB in non-transection trigeminal vessel, perineurium and fasciculus. (D–E) Trigeminal transection reduced EB in perivascular spaces or tissues, in the perineurium and fasciculus. (G–I) EB distributed into perivascular spaces in medulla, pons and pia mater. (J–I) trigeminal transection reduced the EB around the vessel in medulla, pons and pia mater. White arrows: perineurial EB; blue arrows: perivascular EB. The bar is 50 μm, 20×; (B) and (E) 40×.

EB diffused along trigeminal and brain vasculatures, perivascular spaces or tissues in the epineurium, perineurium, dura mater and pia mater (Figures 2 and 4). The rostral trigeminal transection reduced epineurial and perineurial EB in caudal trigeminal (Figure 5(A–F)). EB around the perivascular spaces but not the endothelium was decreased (Figure 5(A,D), blue arrows). Trigeminal transection decreased epineurial and perineurial EB (Figure 5(B,E), white arrows). Transection reduced EB in the fasciculus (Figure 5(B,E), white arrowheads) and attenuated ring-like EB in the caudal trigeminal nerve (Figure 5(C,F), white dash circles), indicating that EB also diffused via fasciculus and Schwann cells. Trigeminal transection attenuated perivascular EB and neuronal EB in medulla oblongata and pons (Figure 5(G,H,J,K), blue arrows and yellow arrows, respectively). Trigeminal transection also reduced EB in pia mater and the sub-pia tissue (Figure 5(I,L). In consideration of trigeminal perivascular spaces or tissues involved in the delivery of EB, EB diffused into the brain perivascular spaces or tissues via the trigeminal perivascular spaces or tissues.

### Dermal perivascular spaces or tissues were involved in the delivery with i.d

**Figure 6.**
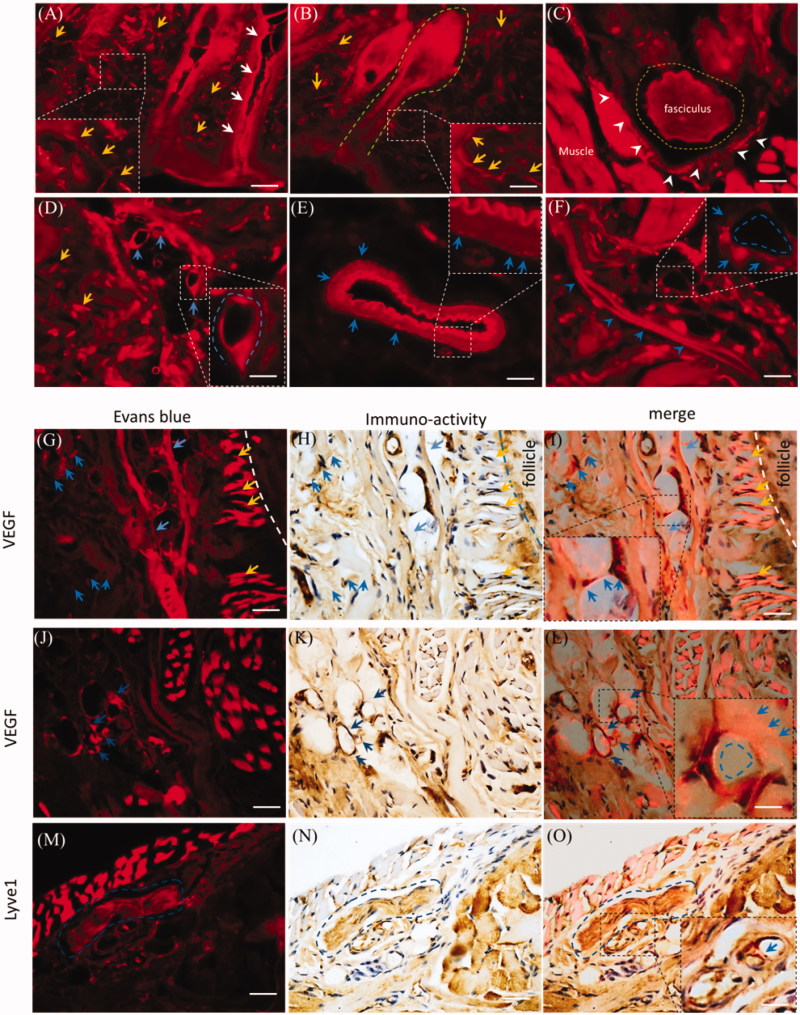
Dermal EB distributed along vasculatures and the perivascular spaces. The EB distribution was observed in the mystacial pad at 2.0 h-post injection and the sections from edge of injection site. (A) and (B) Evans blue diffused into the mystacial sweat glands and hair follicles. (C) EB distributed in the fasciculus and the perineurium. (D) and (E) Evans blue diffused along endothelial cells and perivascular tissues. (F) Evans blue distributed along the lymphatic vessel and blood capillary. (G–L) EB overlaid or surrounded VEGF immuno-reactivity. (M–O) EB located around the Lyve1 immuno-reactivity. White arrows: the sweat gland; green dash line: hair follicle; yellow arrows or dash circle: the sensory nerve fibers and fasciculus; blue arrows or dash circles: vasculatures and perivascular spaces or tissues; white arrowheads: the perineurium. The bar is 50 μm, (A–F) 20×, (G–O) 40×.

Trigeminal and brain perivascular and lymphatic EB might derive from the mystacial pad (Figures 3 and 5). With i.d., EB diffused in all sub-structures of the mystacial pad, such as the sweat gland, hair follicles, neurons, vasculatures and muscles (Figure 6(A–F)). EB diffused along the sweat gland and hair follicles (Figure 6(A,B), white arrows and dash green line). EB distributed into the sensory nerve fibers (Figure 6(A,B), yellow arrows), the fasciculus (Figure 6(C), dash yellow circle) and the incomplete perineurium (Figure 6(C), white arrowheads). In the mystacial pad, EB also distributed into vasculatures and the perivascular spaces or tissues as well as in trigeminal nerve. For instance, EB diffused into the lumen and stained the endothelial cells (Figure 6(D,E), blue arrows) and the outmost layer and surrounding tissue of blood vessels (Figure 6(E), blue arrows). In addition, after i.d., EB stained the lymphatic vessel (Figure 6(F), blue arrowheads) and capillary (Figure 6(F), blue dash circle). Dermal EB overlaid or surrounded the VEGF immuno-reactivity (Figure 6(G–L)), blue arrows and dash circle) and capillary (Figure 6(G–L)), yellow arrows), indicating the blood vasculatures and perivascular tissues might be involved in the EB diffusion. Moreover, EB also overlaid the Lyve1 immuno-reactivity (Figure 6(M–O)), blue arrows) and fasciculus in mysticial pad (Figure 6(M–O)), blue dash circle). It suggested the perivascular spaces or tissues and the lymphatic vessels might be involved into EB delivery.

## Discussion

In the present study, using the dye EB as tool, we demonstrated the dermal-trigeminal-brain perivascular spaces or tissues were main pathways for intradermal brain-targeted delivery, and the lymphatic network linked to perivascular spaces or tissues. EB was extensively used as lymphatic vessels tracer in CSF drainage and the BBB permeability; in addition, EB binds to extracellular matrix, such as collagen, elastin and AMPA receptors (Heinle and Lindner, 1984; Weiser et al., 1996; Marchi et al., 2010; Maloveska et al., 2018). It is easy to visualize the structures or tissues and the lymphatic vessels. Free EB and the albumin-binding EB could not penetrate into brain unless the BBB opening. Therefore, EB in perivascular tissues or brain parenchyma is derived from dermal EB via sub-trigeminal structures rather leakage of the blood vessels. Pre-experimental data (data not shown) showed EB at 4% (20 μL/10g bodyweight) i.d. induced the lethal toxicity in the mice while EB i.v. did not, indicating the intradermal injection delivered more EB to the brain. Thus, EB at lower dose, 5 mg/kg for mice and 7.1 mg/kg for rat without toxicity, were selected for visualization of the delivery pathways.

Brain perivascular spaces or tissues functioned as pathway for intradermal delivery but not drainage from brain for EB. In CNS, lacking of lymphatic system in brain parenchyma, the perivascular spaces or tissues functioned as the channel for CSF drainage and drugs diffusion into the parenchyma (Yang et al., 2013; Yin et al., 2013; Reiter et al., 2014; Lochhead et al., 2015; Dobson et al., 2017; Mestre et al., 2017). The vascular basement membrane was also viewed as pathways for fluid in and out (Morris et al., 2016). Recently, the perivascular spaces or tissues were viewed as the most efficient pathway for CSF influx in the midbrain after gadopentetate dimeglumine being injected into cisterna magna (Dobson et al., 2017). EB with i.d. occurred in the caudal brain, especially the brain stem (Figure 1(A,B)). In consideration of the trigeminal nerve, a nerve connected to brainstem, was the main pathway for facial intradermal brain-targeted delivery (Yu et al., 2017). Thus, EB with i.d. might diffuse into brain perivascular spaces or tissues but not drainage from the brain parenchyma.

The trigeminal perivascular spaces or tissues functioned as pathways for facial intradermal brain-targeted delivery. Trigeminal EB occurred along the perineurial and epineurial perivascular spaces and lumen after i.d., and EB were intense than other sub-trigeminal tissues (Figure 2). The dura and pia mater elongated to the perineurium and epineurium, respectively. A microcirculatory bed occurs in the human trigeminal perineurium (Smoliar et al., 1998). Moreover, the intracerebral arteries or veins were covered by a thin sheath of pia mater, and the perivascular spaces located between vessels and pia mater(Zhang et al., 1990). It suggested trigeminal perivascular spaces or tissues connected to the dural and leptomeningeal perivascular spaces or tissues.

 We found EB diffusion along the brain midline (Figure 1(B)), ependymal, dura and pia mater (Figure 4(F,M–O)). Our previous data also indicated the higher intensity of EB in ependymal and choroid (Yu et al., 2017). The pia mater occurs in the deep brain, and folds inward to form the choroid and choroid plexuses of the ventricles (Adeeb et al., 2013). It might be a pathway for macromolecules and particles delivery (Papisov et al., 2013). The intracerebral perivascular spaces located between vessels and pia mater(Zhang et al., 1990). It indicated EB with i.d. might be delivered into brain via perivascular spaces along the ependymal, choroid and brain vasculatures. The *in-vivo* imaging indicated EB with i.d. delivered into rostral trigeminal end (Figure 1(C)), and trigeminal transection reduced trigeminal epineurial, perineurial and brain EB (Figure 5). In addition, perivascular spaces were involved in rapid intranasal brain-targeted delivery (Lochhead et al., 2015) and substances diffusion in brain parenchymal (He et al., 2012; Yin et al., 2013). Thus, trigeminal perivascular spaces or tissues bridged to the skin and brain, dermal-trigeminal-brain mediated the intradermal brain-targeted delivery of EB.

The lymphatic network linked to perivascular spaces or tissues, such as basement membrane. In the present study, Lyve1 immuno-reactivity located beside the vessels linked to trigeminal perivascular tissues or spaces, and overlaid EB (Figure 3(D–N)), demonstrating the lymphatic network connected to perivascular spaces. Lymphatic vessels travel along trigeminal nerve (Mohan et al., 2009), and the lymphatic network in the dura mater of the mouse brain served as a pathway for drainage of CSF and macromolecules (Aspelund et al., 2015). There might be a communication between lymphatic network and perivascular spaces or tissues for EB delivery after facial intradermal brain-targeted injection.

Trigeminal EB might derive from mystacial lymphatic network and nasal lymphatic network mainly. The nasal and trigeminal lymphatic vessels linked to subarachnoid space and served as rapid pathways for intranasal delivery (Thorne et al., 2004; Johnston et al., 2007; Yang et al., 2013; Lochhead et al., 2015), and nasal lymphatic vessels linked to perivascular spaces or tissues (Zakharov et al., 2003). The nasal lymphatic system was involved in intranasal brain-targeted drug delivery (Thorne and Frey, 2001; Thorne et al., 2004; Hanson and Frey, 2007; Dhuria et al., 2010; Kim et al., 2014). Our previous work indicated the mystacial lymphatic network linked to nasal lymphatic network (Yu et al., 2017). In addition, EB distributed along or around the dermal vasculatures, perivascular spaces and tissues after i.d. (Figure 6(A–E)). In consideration of the lymphatic network linked to perivascular spaces or tissues, dermal EB diffused into brain via lymphatic network-nasal perivascular spaces or tissues.

The dermal lymphatic network also connected to trigeminal nerve directly. The dermal lymphatic network involved in the drug absorption after skin administration, and lymphatic network inhibitor i.d. or i.n. disturbed the EB diffusion in trigeminal nerve (Jain et al., 2008; Harvey et al., 2011; Yu et al., 2017). After i.d., EB entered the lymphatic vessels and submandibular lymph nodes (Figure 3(N–P)), demonstrating the EB diffusion into lymphatic systems. EB around the blood capillary was lower than in lymphatic vessel (Figure 6(F)). EB overlaid VEGF immune-activity (Figure 6(G–L)), EB col-located with Lyve1 immuno-reactivity beside the fasciculus (Figure 6(M–O)), indicating vasculatures, the perivascular spaces or tissues and lymphatic network were involved in the EB delivery along the nerve, perivascular spaces or tissues. Thus, the dermal lymphatic network mediated the delivery of EB from mystacial pad to trigeminal nerve via vasculatures and perivascular spaces or tissues. This should be clarified in the future.

## Conclusions

In the present study, facial intradermal injection in mystacial pad, intranasal and intravenous injection delivered EB into the mouse brain in different style. Trigeminal perivascular spaces or tissues bridged to dermal and brain perivascular spaces or tissues, and dermal-trigeminal-brain perivascular spaces or tissues served as pathways for facial intradermal brain-targeted delivery. The perivascular spaces or tissues linked to lymphatic network. These findings demonstrated the dermal-trigeminal-brain perivascular spaces or tissues mediated the facial intradermal brain-targeted delivery. The lymphatic communication with perivascular spaces or tissues was involved in the facial intradermal brain-targeted delivery.
